# *Astragalus* Polysaccharide Extends Lifespan via Mitigating Endoplasmic Reticulum Stress in the Silkworm, *Bombyx mori*

**DOI:** 10.14336/AD.2019.0515

**Published:** 2019-12-01

**Authors:** Jiangbo Song, Min Chen, Zhiquan Li, Jianfei Zhang, Hai Hu, Xiaoling Tong, Fangyin Dai

**Affiliations:** State Key Laboratory of Silkworm Genome Biology, Key Laboratory for Sericulture Biology and Genetic Breeding, Ministry of Agriculture and Rural Affairs, College of Biotechnology, Southwest University, Chongqing 400716, China

**Keywords:** *astragalus* polysaccharide, silkworm, experimental animal, drug efficacy, lifespan, endoplasmic reticulum stress

## Abstract

The traditional Chinese medicine *Astragalus* polysaccharide (APS) has been widely used to improve glucose homeostasis and immunoregulator properties. In recent years, it has also been shown to extend the lifespan of *Caenorhabditis elegans*; however, the underlying molecular mechanisms are not fully understood. Here, our study shows that APS could significantly extend adult stage, mean, and maximum lifespan of the silkworm, *Bombyx mori* and increase body weight without affecting food intake and fecundity. Meanwhile, the activities of glutathione S-transferase and superoxide dismutase are significantly enhanced, and the reaction oxygen species content is reduced concomitantly. Moreover, the activity of lysozyme is increased dramatically. In addition, APS rescues the shortened lifespan by *Bacillus thuringiensis* infection in silkworm. Furthermore, the transcription of the crucial genes involved in endoplasmic reticulum stress is upregulated upon the endoplasmic reticulum stress stimulation. APS also significantly ameliorates endoplasmic reticulum stress in silkworm cell line and in vivo. Together, the results of this study indicate that APS can prolong the silkworm lifespan by mitigating endoplasmic reticulum stress. This study improves our understanding of the molecular mechanism of APS-induced lifespan extension and highlights the importance of the silkworm as an experimental animal for evaluating the effects and revealing the mechanisms in lifespan extension of traditional Chinese medicine.

Radix Astragali (the root of *Astragalus* propinquus) is a widely-used herb in the traditional Chinese medicine (TCM) with a long history [1]. In western herbal medicine, Radix Astragali has been used for the treatments of immune system disorders [2], viral infections [3, 4], insulin sensitivity [5], neurotoxicity [6, 7], cancer [8-10] and aging [11, 12]. APS is the polysaccharide component and major active component of *Astragalus* and a commonly used nutraceutical with lifespan-extending capabilities [1]. It improves whole-body glucose homeostasis by activating AMPK [13] and attenuating endoplasmic reticulum stress (ERS) and affecting insulin/insulin-like growth factor-1 signaling (IIS) pathways, which are associated with longevity [14]. Notably, it was recently reported that APS could extend the lifespan of *Caenorhabditis elegans* (*C. elegans*) independent of DAF-2/insulin-like receptor but dependent on the DAF-16/FoxO transcription factor [7]. Interestingly, APS has also been shown to prolong the lifespan of *C. elegans* by increasing the level of miR-124, which in turn, decreased the expression of activating transcription factor-6 (ATF6) [15]. However, the precise pharmacological mechanisms by which APS extends lifespan are still poorly understood.

Cells respond to accumulation of misfolded proteins in the ER by activating the unfolded protein response (UPR) signaling pathway [16]. The ERS response consists of three signaling pathways [17], which are mediated upstream by ATF6, protein kinase R-like ER kinase (PERK), and inositol-requiring enzyme 1 (IRE1), three ER-resident transmembrane proteins. Normally, these three upstream mediators bind to ER partner-bound immunoglobulin (Bip) [17], which encodes a major chaperone protein and is often considered a marker for ERS [18]. Thapsigargin, an ERS inducer, induces the transcription of various conventional ERS genes, especially Bip. ERS and protein aggregation have been implicated in aging [19]. Reducing ERS promotes correct protein folding, enhances efficiency of ER molecular chaperones, and can delay aging and prevent the occurrence of some age-related diseases. The UPR restores ER homeostasis by degrading misfolded proteins, inhibiting translation, and increasing the expression of chaperones that enhance ER protein folding. The efficiency of UPR signaling declines during aging [20]. Previous research has shown that improving ERS tolerance can extend the lifespan of different model organisms [21, 22]. Furthermore, it has been shown that deletion of UPR target genes can significantly prolong lifespan [20]. It has been shown that a loss of function mutant in the insulin signaling pathway with activated FoxO plays a role in lifespan extension [23, 24]. The ERS-sensing proteins IRE-1 and X-box binding protein 1 are involved in longevity of insulin/insulin growth factor-1 pathway mutants [25]. These studies revealed that the UPR signaling pathway is directly related to lifespan.

As a typical representative of Lepidopteran insects, the silkworm *Bombyx mori* has numerous advantages and convenience as an experimental animal model for evaluating the effects of TCMs [26-30].

## MATERIALS AND METHODS

### Silkworm strain and feeding conditions

For background controls, we used an inbred line (N4; Silkworm Gene Bank of Southwest University, Chongqing, China). Silkworms were maintained at 25°C in approximately 75% relative humidity with a 12L:12D photoperiod during the whole life cycle, and were reared on an artificial diet (Silkmate 2S; Nosan Corporation, Yokohama, Japan) during the whole larval stage [31]. The APS was dissolved in water and mixed with the artificial diet. The artificial diet with or without APS was autoclaved at 98°C for 25 min.

### Reagents

APS (in powder form) was a commercial product (purity: 70 %) purchased from Shanghai Canspec Scientific Instruments (Shanghai, China). Thapsigargin was purchased from Sigma (T9033, St. Louis, MO, USA). Glutathione S-transferase (GST) Assay Kit, Superoxide Dismutase (SOD) Assay Kit, malondialdehyde (MDA) Content Assay Kit, and Reactive Oxygen Species (ROS) Assay Kit were purchased from Suzhou Comin Biotechnology (Suzhou, China). The silkworm Attacin ELISA Kit and Silkworm Lysozyme ELISA Kit were purchased from Xiamen Huijia Biotechnology (Xiamen, China).

### Cells and pathogens

The *Bombyx mori* ovary cell line BmN-SWU1 (BmNS) was cultured at 27°C in TC-100 medium (United States Biological, Salem, MA, USA) supplemented with 10% (v/v) fetal bovine serum (Gibco, Gaithersburg, MD, USA) and 2% (v/v) penicillin/streptomycin (Gibco, Gaithersburg, MD, USA). *Bacillus thuringiensis* (BT) is maintained in our laboratory.

### Cell proliferation assay

Silkworm BmNS cells were seeded in 12-well cell culture plates at a cell density of 1.5×10^5^ cells/mL TC100 medium and cultured for 24 h. Then the medium was replaced with medium containing 0.2 μg/mL Thapsigargin, followed by incubation for 6 h. Then the cells were washed twice with PBS and incubated with medium containing 400 μg/mL APS for 12 h. Finally, 10% CCK solution was added to the cell culture medium. After incubation for 2 h, the absorption at 450 nm was detected with a microplate analyzer.

### Silkworm lifespan assay

All lifespan experiments were performed at 25°C on silkworms fed an artificial diet containing no APS as a control or 0.1% (1g/kg of diet) APS as treatment. Lifespan was monitored every 3 h from day 1 of the adult stage. Silkworms that did not move when gently prodded were marked as dead and recorded. Maximum lifespan refers to the upper 10% of the distribution of lifespan.

### Daily food intake, body weight, and fecundity assays

The 5^th^ instar larvae were randomly chosen; there were 20 silkworms per group, and daily food intake, body weight, and fecundity were measured. The food supply and residual amount were measured daily to calculate the mean amount of food intake. Body weight was also measured daily at the same time. The number of progenies per female moth was counted. To determine fertility of silkworm, 20 silkworms at the beginning of the egg-laying period were placed on plates and removed after 1 day. Offspring were allowed to develop for 8 days, and then infertile eggs and hatched larvae were quantified.

### SOD, GST, ROS, and MDA assays

To evaluate the drug effects in a timely manner, antioxidative assays, immunological parameters, and the antibacterial capability test were performed at the larval stage. The levels of SOD and GST and the contents of MDA and ROS were measured using test kits (SOD, GST,

MDA content, and ROS content assay kits). The activities of SOD and GST and contents of MDA and ROS in the whole-body homogenate were measured according to the kit’s instructions.

### Immunological parameters assays

Blood samples were collected at day 1, 3, and 5 from 5^th^ instar larva. Blood samples were taken from the third proleg using surgical scissors and were stored at -80°C until measurement of lysozyme and Attacin (Att) content. The levels of lysozyme and antimicrobial peptide were measured using test kits (silkworm Attacin and lysozyme ELISA Kits). The contents of lysozyme and Att were measured according to the kit’s instructions.

### Antibacterial capability test

Overnight cultures of BT were diluted 100-fold with PBS and 0.01 mL was injected into the hemolymph through the spiracle using a glass needle. The injected larvae (day 3 of 5^th^ instar larva) were maintained without food in an artificial climate incubator (RXZ, Ningbo, China) at 25°C with 70% humidity, and survival was monitored for 12 h after injection.

**Table 1:** The primer sets used in this study for RT-qPCR.

Primer name	F primer (5’ - 3’)	R primer (5’ -3’)
sw22934	TTCGTACTGCTCTTCTCG	CAAAGTTGATAGCAATTCCCT
BmATF4	CGTCGCCCTAACCGTATCAT	CCAGTCTTCCACCGCATTCT
BmATF6	GAGTCGTTGGATTAGAGGAGGC	GATGTTACGCACCTGATTTCTTG
BmBip	CCGCCCTTTAACTTTCCACTC	GACACGCTGCCGTCGCTA
BmeIF2α	CATCACAGAGGCAGGTGGAGT	CAGCGAGTTCAGCGTTTTCA
BmPERK	TGGCTTTGGCGTTAGTCTTGTT	CTCGGATGGTATGTCCTCGTT
BmIRE1	GTTGGGCTGCGTGTTCTATTAC	AGAACATCGGGTATTTCAGTATCG
BmFoxO	GCACAGGACAACAGGCTCACAC	GCTTGGCGTCGGGATTGA
BmHSP70	CTCTCGCTGACCAGGATGAAT	TTCGACAGTGGGTCCATCATT
BmHSP90	TCCCAGTTCATTGGCTACCC	TTGACTGCAAGATGGTCCTCC
BmTRAP1	GATTCTCATCCTTGCGTCATTGT	TGTTCGCTAGTTCCTTGTCAGTC

### Real-time quantitative PCR

Total RNA was extracted from the cells or silkworms of the different groups using the Rapid Extraction Total RNA Kit (BioTeke Corporation, Beijing, China), according to the manufacturer’s instructions. cDNA synthesis was done using the PrimeScriptTM RT Reagent Kit with gDNA Eraser (TaKaRa, Dalian, China). For real-time quantitative (RT-qPCR), the cDNA was assessed on the CFX96 Real-Time PCR system (Applied Biosystems, CA, USA) with the iTaq universal SYBR Green Supermix Kit (Applied Biosystems), according to the manufacturer’s protocol. The eukaryotic translation initiation factor 4A (BmMDB probe ID sw22934) was used as the internal reference [32]. The primer pairs are listed in Table 1.

### Statistical analysis

Statistical analyses of the survival curves were performed using the log-rank test (Mantel-Cox) (PRISM software package, GraphPad software). The average of the mean lifespan and the maximum lifespan of a set of independent experiments were calculated and represented as the mean ± standard error of the mean. The log-rank test (Mantel-Cox) method and Student’s *t*-test (two tailed) were employed to perform statistical analysis. *p<*0.05 was considered statistically significant.

## RESULTS

### APS extends the lifespan of the female silkworm

**Figure 1.**
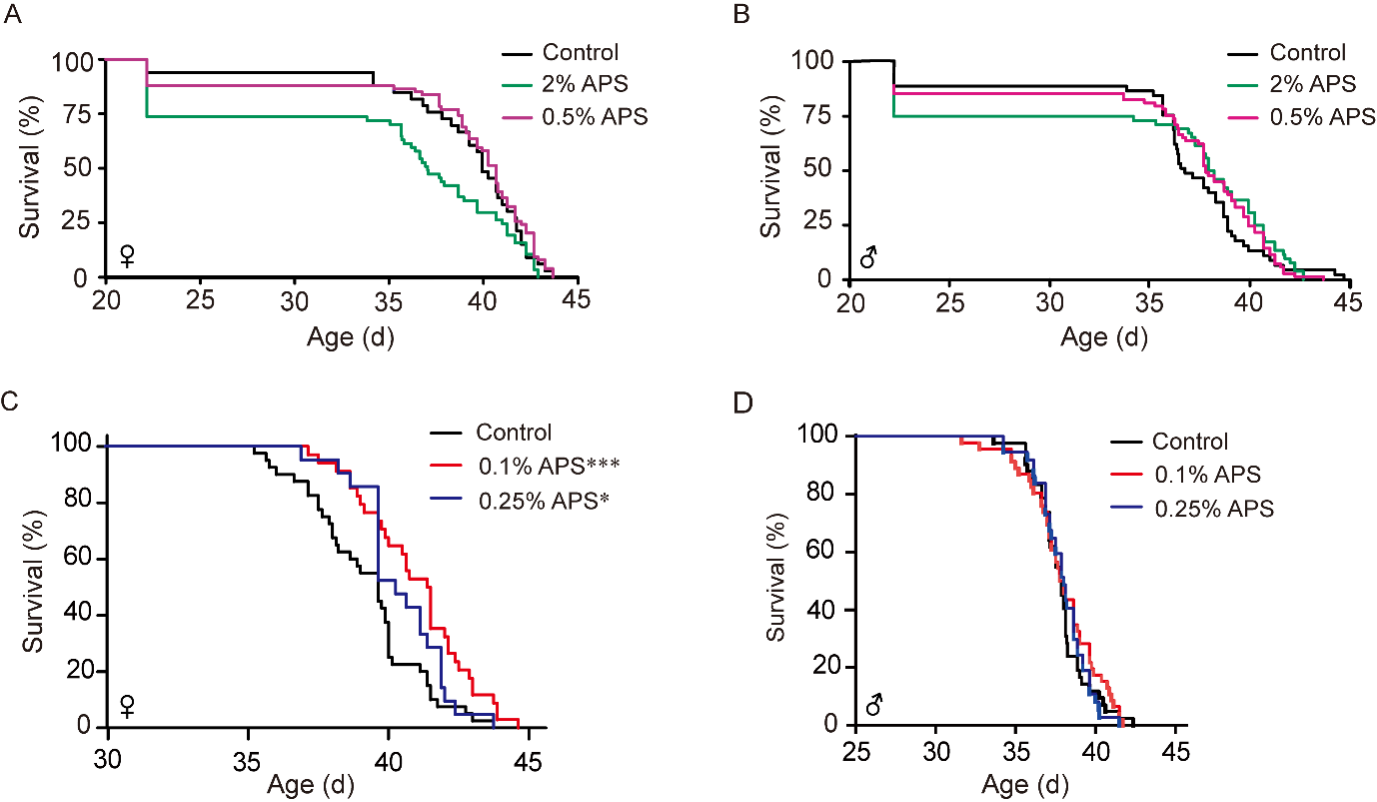
The effective concentration of APS. (A-D) Survival curves of different APS concentrations in female and male silkworms. Log-rank test (Mantel-Cox) and Student’s *t*-test (two tailed) were used to evaluate the difference between the treatment and control groups. *p<0.05. **p<0.01. ***p<0.001.

To detect the suitable concentration on lifespan in silkworm, we performed 2% and 0.5% mass fraction in female and male silkworm. The results showed that 2% APS significantly shortened the mean lifespan of the female silkworm with no obvious effect on the male silkworm. Meanwhile, 2% APS reduced larvae survival, as some silkworms were not able to properly molt and died at the larval stage, while the adult stage of survival individuals was prolonged in the male silkworm. (Fig. 1A, B). Subsequently, the effects of the reduced concentrations of 0.25% and 0.1% APS were explored and found to significantly extend lifespan in the female silkworm but not in the male silkworm. The phenomenon of partially failing to molt disappeared (Fig. 1C, D). The concentration of 0.1% APS had a better lifespan-extending effect than the other concentrations test, so 0.1% APS was used for subsequent studies.

**Figure 2.**
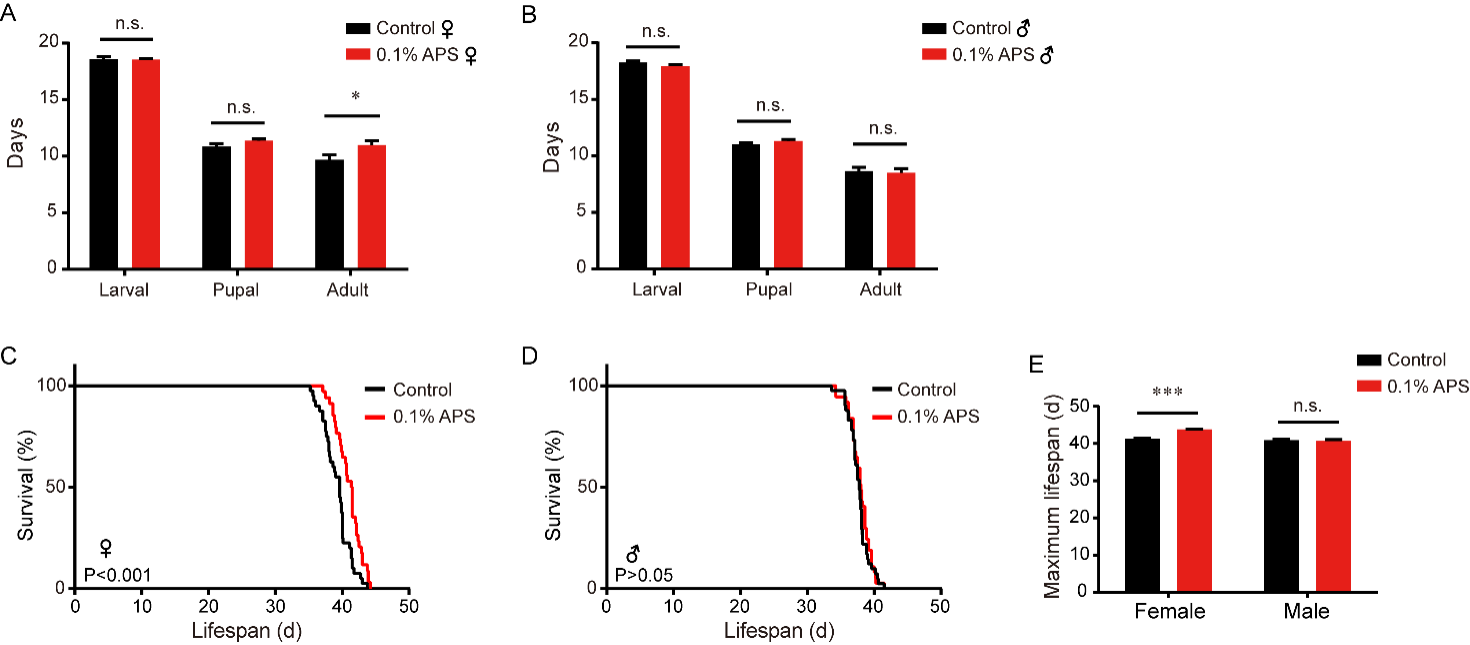
Effect of APS on the lifespan of the silkworm. (A) Unmated females treated with APS (mean lifespan=41.0 d, n=33) lived longer than control female silkworms (mean lifespan= 39.5 d, n=36), *p*<0.001; (B) There was no significant difference between unmated males treated with APS (mean lifespan=37.96 d, n=42) and control male silkworms (mean lifespan=37.72 d, n=37), *p*=0.51. C. Unmated females treated with APS (maximum lifespan=43.79 d, n=4) lived longer than control female silkworms (maximum lifespan=41.28 d, n=4), p=0.0001; (D) There were no significant differences between unmated males treated with APS. (E) The maximum lifespan of treatment and control in female and male silkworm. Log-rank test (Mantel-Cox) and Student’s *t*-test (two tailed) were employed to evaluate the significance between the control and treatment groups. *p<0.05. **p<0.01. ***p<0.001. n.s., not significant.

The larval stage and pupal stage did not significant differ after treatment with 0.1% APS compared to the control while only the adult stage was prolonged (13.3%) in the female silkworm (Fig. 2A). There was also no difference in males among the larval, pupal, and adult stages between the treatment and control groups (Fig. 2B). Moreover, the females in the APS treatment group displayed an extended mean lifespan (40.88 days) compared to the control (39.23 days). Thus, the mean lifespan of APS-treated female silkworms was increased by 4.21% compared to the control (Fig. 2C), but this phenomenon did not occur in males (Fig. 2D). The maximum lifespan of females was extended by 1.1 days (2.66%) (Fig. 2E).

### APS increases body weight without affecting daily food intake and fecundity

**Figure 3.**
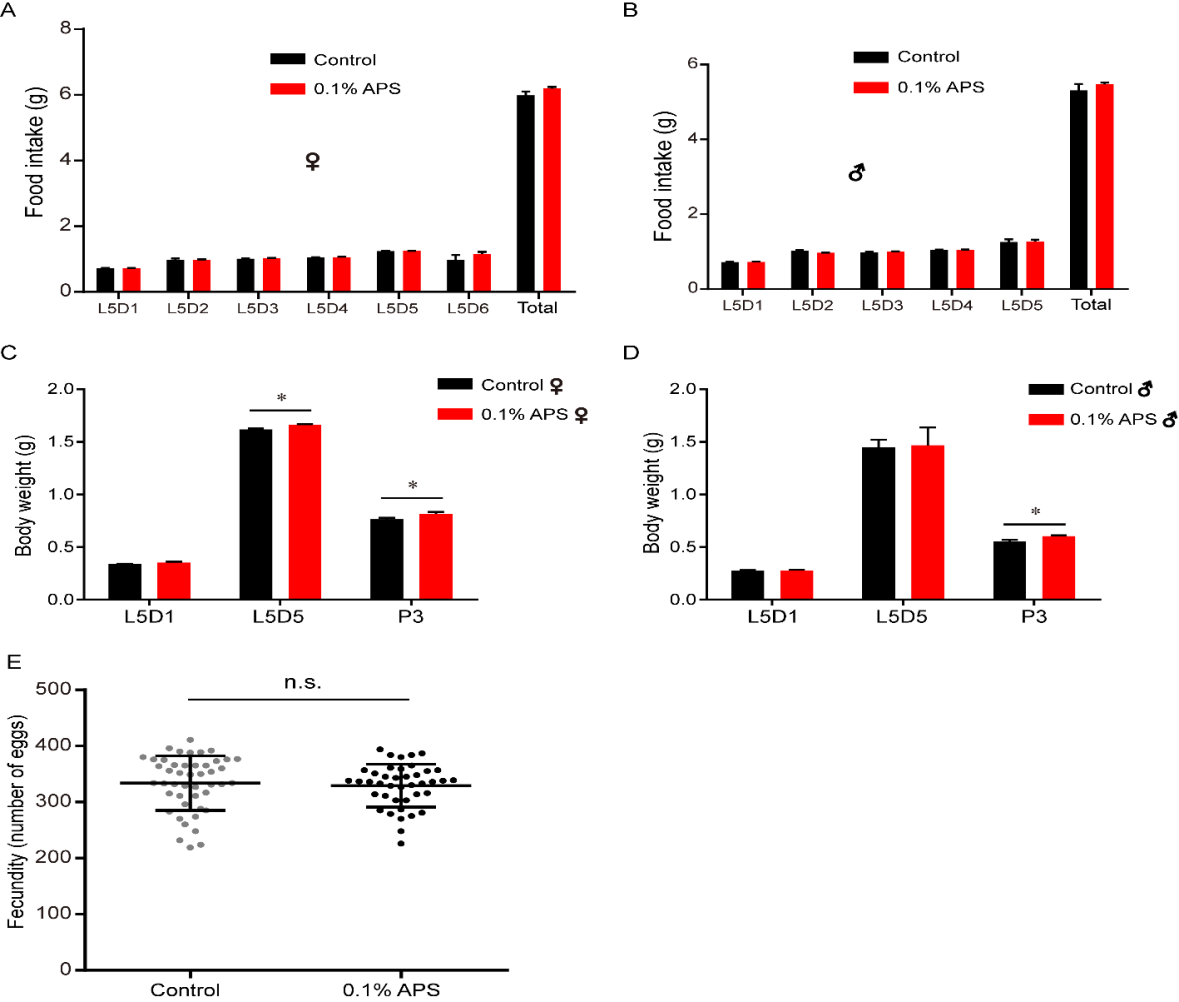
Effect of APS on the diet, weight, and fecundity of the silkworm. (A-B) The impact on food intake of female and male silkworm chronically exposed to APS (n=20); (C) The impact on body weight of female silkworm chronically exposed to APS, L5D1 (mean_Ctrl_ =0.338 g, mean_APS_=0.351 g, *p*=0.306, n=20), L5D5 (mean_Ctrl_ =1.618 g, mean_APS_=1.662 g, *p*=0.025, n=20), P3 (mean_Ctrl_ =0.765 g, mean_APS_=0.816 g, *p*=0.039, n=8); (D) The impact on the body weight of male silkworm chronically exposed to APS or respective controls, L5D1 (mean_Ctrl_ =0.274 g, mean_APS_=0.278 g, *p*=0.69, n=20), L5D5 (mean_Ctrl_ =1.451 g, mean_APS_=1.467 g, *p*=0.93, n=20), P3 (mean_Ctrl_ =0.552 g, mean_APS_=0.604 g, *p*=0.020, n=9). Student’s *t-*test (two tailed) were employed to evaluate the significance. *p<0.05. n.s., not significant. (E) There were no significant differences between females treated with APS (fecundity =329, n=41) and control female silkworms (fecundity =333, n=46), p=0.635. Student’s *t-*test (two tailed) were employed to evaluate the significance. n.s., not significant.

Caloric restriction or reduced food intake increases the lifespan of many organisms [33-35]. Reproductive output is often negatively correlated with lifespan, and repression of fecundity is general sufficient to extend the lifespan [36]. To determine whether the observed lifespan extension following APS treatment was from energy exchange, we examined the food intake, body weight, and fecundity in silkworms with or without APS treatment. The results showed that the daily food intake of 5^th^ instar larvae with 0.1% APS treatment was similar to that in the control female and male silkworms (Fig. 3A, B), indicating that daily food intake did not play a role in the observed lifespan extension. Interestingly, we found that APS increased the body weight of both sexes with similar daily food intake (Fig. 3C, D). We speculate that APS may impact the metabolic rate of silkworms, resulting in the different conversion of energy substances with the same food intake in different groups.

We also examined the reproductive performance of silkworms in the treatment and control groups and found that both groups had similar fecundity (Fig. 3E). The uncoupling between lifespan and reproduction suggests that fecundity may not be relevant to the extended lifespan induced by APS.

### APS enhances antioxidative properties in silkworm

**Figure 4.**
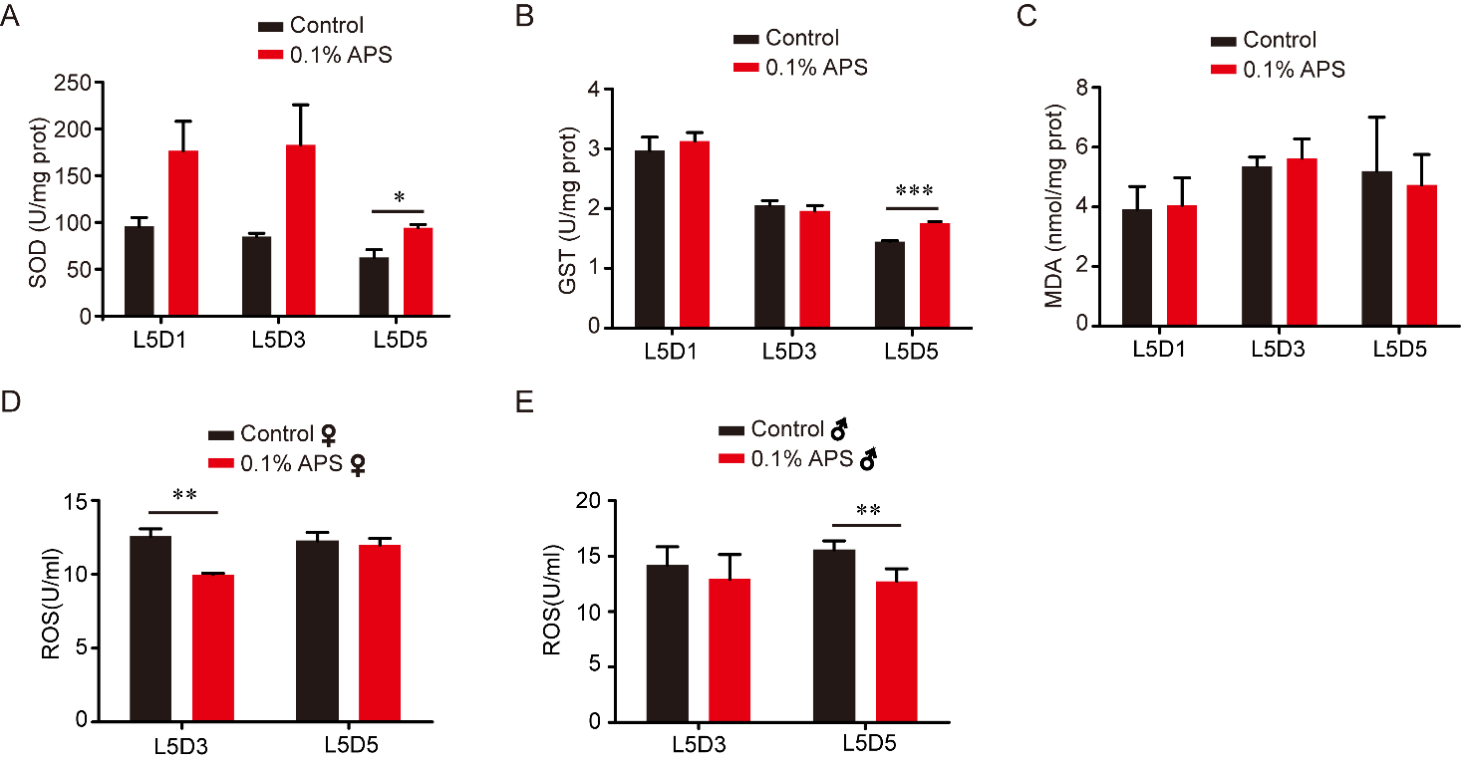
Effects of APS on SOD and GST activity, and the content of MDA and ROS in the silkworm. (A) The standard curves of the SOD, the SOD activity of the fifth larval silkworm after 0.1% APS chronically treatment, L5D1 (Student’s *t*-test, two-tailed, n=9), L5D3 (Student’s *t*-test, two-tailed, n=9), L5D5 (Student’s *t*-test, two-tailed, n=9). (B) The standard curves of the GST, the GST activity of the fifth larval silkworm after 0.1% APS chronically treatment, L5D1 (Student’s *t*-test, two-tailed, n=9), L5D3 (Student’s *t*-test, two-tailed, n=9), L5D5 (Student’s *t*-test, two-tailed, n=9). (C) The standard curves of the MDA, the MDA content of the fifth larval silkworm after 0.1% APS chronically treatment, L5D1 (Student’s *t*-test, two-tailed, n=9), L5D3 (Student’s *t*-test, two-tailed, n=9), L5D5 (Student’s *t*-test, two-tailed, n=9). (D-E) The ROS content of the fifth larval silkworm after 0.1% APS chronically treatment, L5D3 (Student’s *t*-test, two-tailed, n=9), L5D5 (Student’s *t*-test, two-tailed, n=9).

Aging is a result of the long-term accumulation of stress [34, 37-39]. The free radical theory holds that the balance between oxidants and antioxidants in organisms and cells play a significant role in aging [40-42]. In addition, the antioxidant capability of an organism is an important component of anti-aging. To determine whether APS extends silkworm lifespan by strengthening its antioxidant ability, we evaluated the major antioxidant defense systems in silkworm. The activity of SOD and GST, and the contents of MDA and ROS are the generally accepted indicators to reflect antioxidant capacity. Hence, we investigated the activity of SOD and GST, and the content of MDA and ROS in the whole body at L5D1, L5D3, and L5D5 in silkworms treated with or without 0.1% APS. Our results showed that SOD and GST activity were significantly increased at L5D5, but there was no significant change at L5D1 and L5D3 (Fig. 4A, B). Compared with the control, the whole-body MDA content did not change at the detected stages (Fig. 4C), whereas the ROS content was decreased at L5D3 in female and at L5D5 in male silkworm (Fig. 4D, E). These results demonstrate that APS could enhance antioxidative ability in the silkworm.

### APS improves immunity in silkworm

**Figure 5.**
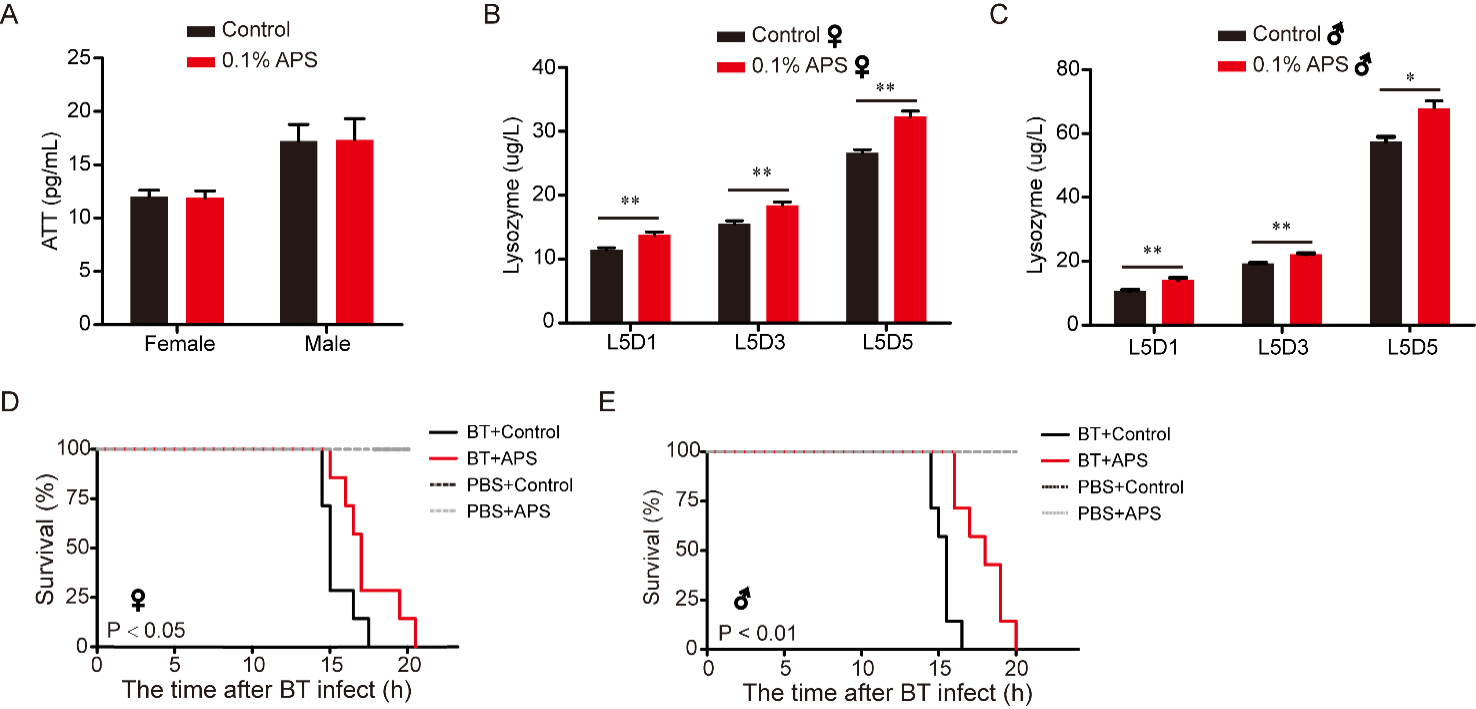
Effect of APS on Att and lysozyme content, and survival condition in the silkworm. (A) Attacin content on day 3 of fifth instar larvae silkworm hemolymph after 0.1% APS chronic treatment, female (Student’s *t*-test, two-tailed, n=7), male (Student’s *t*-test, two-tailed, n=7). (B and C) Lysozyme content on day 3 of fifth instar larvae silkworm hemolymph after 0.1% APS chronic treatment in female and males (Student’s *t*-test, two-tailed, n=7); (D and E) Survival rates of *Bacillus thuringiensis* (BT)-infected silkworms after chronic exposures to APS, Log-rank test (Mantel-Cox), Female (n=7), Male (n=7).

It has been reported that APS may function as an immunomodulator, inducing changes in the expression of immune response genes [2, 43]. The immune system is a critical component for maintaining health, whereas aging is characterized by the functional decline of the immune system [44]. Therefore, we investigated the effects of APS on the content of the main innate immune parameters in the silkworm. The results showed that there was no significant difference in Att content between the two groups (Fig. 5A). The alkaline lysozyme could eliminates pathogenic microorganisms by catalyzing the cell wall of bacteria into soluble glycopeptides [45]. Here, APS significantly increased the lysozyme content in the blood of 5^th^ instar larvae (Fig. 5B, C). Furthermore, to analyze the difference in immune capacity between the APS-treated group and control, we analyzed the percent survival of silkworms after infection with BT with or without APS, and found that BT acutely shortened the survival rate while APS reversed the shortened survival rate in female and male silkworms (Fig. 5D, E). By comparing the immune capacity between the APS-treated and control groups, we found that the mean lifespan of female and male individuals in the treatment group (19.6 and 15.4 h, respectively) was longer than that in the control group (17.8 and 13.1 h, respectively). Meanwhile, APS supplementation significantly increased resistance to pathogenic microorganisms (Fig. 5). These results suggest that APS may improve the immunity of the silkworm.

### APS alleviates ERS in silkworm cells and silkworm

**Figure 6.**
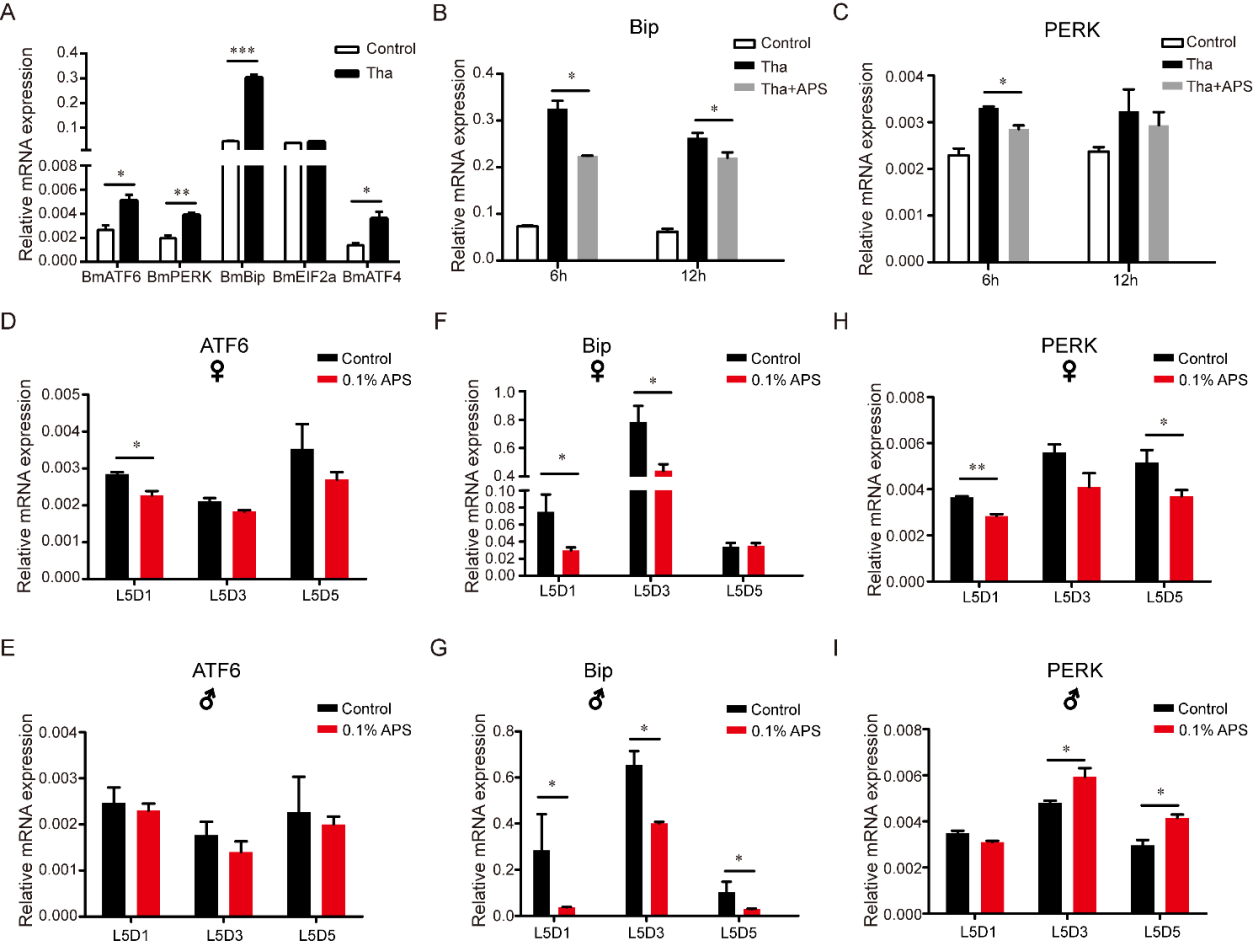
Effects of APS on thapsigargin-induced ERS in BmN-SWU1 cells. (A) Expression levels of ERS-related genes in BmN-SWU1 cells treated with thapsigargin (n=6); (B and C) Effects of APS on the expression levels of *BmBip* and *BmPERK* in BmN-SWU1 cells after thapsigargin treatment (n=6); (D-I) Effects of APS on the expression levels of *BmATF6*, *BmBip*, and *BmPERK* in female and male silkworms (n=9). Student’s *t*-test, two-tailed, *** *p*<0.001; ** *p*<0.01; *p<0.05.

Our results showed that APS could expand lifespan and enhance innate immunity. A previous study suggested that the ER may be the key organelle for longevity and that there is a close relationship among ERS, antioxidant capacity, immunity, and aging [19]. In addition, APS has been shown to restore glucose homeostasis by affecting ERS and IIS pathways, which are associated with longevity [14, 46, 47]. Based upon previous research, we hypothesized that APS may have the ability to regulate ERS, which can be evoked by thapsigargin in animal cells [48]. To determine the effects of APS on ERS in the silkworm, BmNS cells were treated with thapsigargin (0.2 µg/mL). Then, the relative expression level of ERS response genes and cell proliferation activity in BmNS cells treated with or without thapsigargin were evaluated. We found that thapsigargin induced the transcription of a number of conventional ERS marker genes, including *BmATF6, BmPERK, BmBip, BmEIF2α and BmATF4* (Fig. 6A). To evaluate the effects of thapsigargin and APS on silkworm cells, we measured cell proliferation activity after 6 h of thapsigargin treatment and co-treatment with thapsigargin and APS, but no significant difference in cell proliferation was found (supplementary Fig. 1A).

There is a close relationship among APS, ERS, and the aging process [19]. APS restores glucose homeostasis by affecting ERS and IIS pathways which is associated with the longevity [14, 46, 47]. Based on previous researches, we hypothesized that APS maybe have the ability to regulate ER stress in silkworm. To determine whether APS treatment influences the ERS response, APS (400 mg/mL) was used to treat BmNS cells that had been treated with thapsigargin for 6 h. The thapsigargin (0.2 µg/mL) treated group was the ERS positive control. The results showed that *BmBip*, *BmPERK*, and *BmIRE1* were significantly decreased after APS and thapsigargin co-treatment compared to treatment with thapsigargin alone (Fig. 6B, C, supplementary Fig. 1B). Expression of the mitochondrial misfolding protein loading indicator (BmTRAP1) and cytoplasm loading indicator (BmHSP70 and BmHSP90) did not change after thapsigargin and APS co-treatment after in the BmNS cell line (supplementary Fig. 1C-E). In addition, we investigated whether the lifespan-regulating factor FoxO participates in the process of APS-inducing longevity and found that no significant difference in thapsigargin treatment and in APS replenishment after Thapsigargin treatment (supplementary Fig. 1F). These results demonstrate that APS significantly relieved the ERS by activating the ER UPR in vitro.

To further investigate the relationship between APS and ERS, we determined the expression levels of *BmBip* and *BmPERK* in silkworm individuals. The *ATF6* expression level did not significantly change in both sexes except at L5D1 in the female silkworm (Fig. 6D, E). The mRNA levels of BmBip were significantly decreased in the 5^th^ instar larvae of both sexes with APS treatment compared to the control (Fig. 6F, G). The *BmPERK* gene, which limits *Drosophila* lifespan by promoting intestinal stem cell proliferation [49], was significantly decreased at L5D1 and L5D5 in the female silkworm with APS treatment compared to the controls (Fig. 6H), suggesting that *BmPERK* is involved in the APS-mediated lifespan extension. The abovementioned results showed that APS significantly relieved ERS and balanced the ER homeostasis in vivo and in vitro (Fig. 6).

## DISCUSSION

In the normal developmental stage of eukaryote, the processes of protein synthesis in the ER are highly efficient and ordered. However, in the aging process, protein synthesis declined efficiently. Redundant proteins and misfolded proteins may accumulate when the ability of protein synthesize of ER exceeds the requirement, which lead the normal function of organelle into dysfunction, so that cell will activate the UPR signaling pathway to eliminate misfolded proteins. Recent studies found that abnormal ERS and UPR signaling pathways are two key components underlying the pathogenesis of age-related diseases and aging. In *Drosophila melanogaster*, knocking down *PERK* in intestinal stem cells is sufficient to promote intestinal homeostasis and extend lifespan [49]. Consistent with this, the mRNA levels of *BmPERK* and *BmBip*, which are required for proper protein folding, were reduced in female silkworms chronically exposed to APS. Combined with the results of antioxidative capacity and immunity, we speculate that the physiological mechanism of APS-inducing lifespan extension is by enhancing antioxidative capacity and immunity in the silkworm.

**Figure 7.**
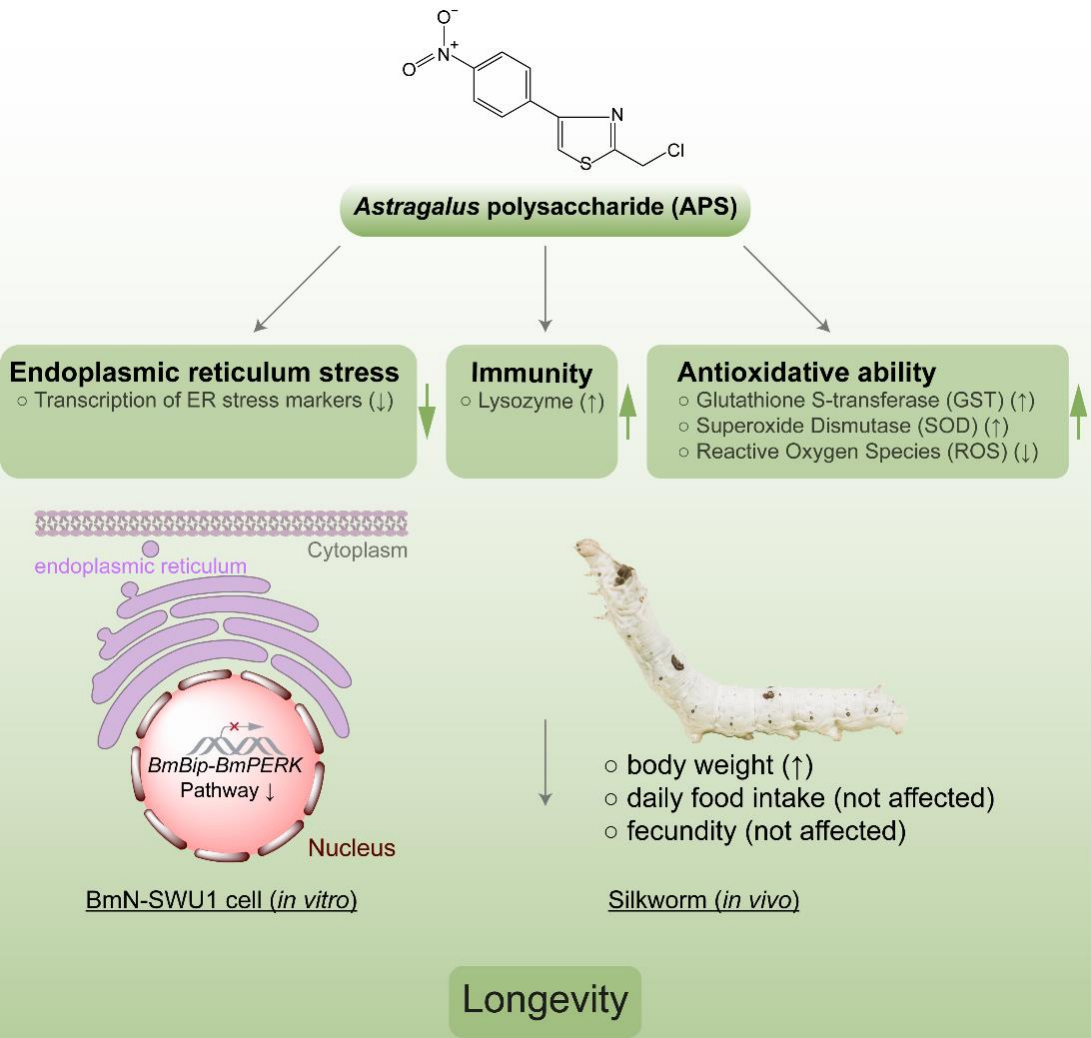
A model illustrating that APS extends lifespan via mitigating ER homeostasis by restricting Bip-PERK pathway activity.

In aging, there are a large number of events that easily trigger ERS such as oxidative stress, protein misfolding and aggregation [50]. However, similar to many other signaling pathways, the UPR suffers from age-related impairment and becomes less effective. The key ER resident chaperones such as Bip required for proper protein folding are impaired during the aging process. We thus speculated that an imbalance of homeostasis exists chronically in the ER with aging. Our results showed that APS could enhance the silkworm’s ability to reduce ERS and balance the homeostasis of the ER in vitro and in vivo. Therefore, the molecular mechanism of APS-mediated lifespan extension is actualized through balancing the ER homeostasis by the Bip-PERK signaling pathway in the silkworm (Fig. 7).

This study demonstrated that 0.1% APS only extended the lifespan of the female silkworm but had no effects on males. Previous research has showed that the lifespan expectancy in females is greater than that in males [31]. The phenomenon was observed in this study as well. A recent research study indicated that the XX genotype increases survival in aging and suggested a protective effect of the ovaries [51]. We hypothesized that there are gender-specific components and molecular pathways located downstream of the XX genotype-induced longevity regulation pathway, which respond to APS induction. It will be of great value to study the mechanism of the different effects of APS on lifespan in both genders.

Using lower animals instead of higher animals to complete preliminary drug testing is developmental trend in the utilization of experimental animals. Numerous advantages make the silkworm a promising model organism for research of aging and drug efficacy evaluation [30]. In this study, silkworm was employed as an experimental animal to estimate the effects of APS on lifespan; we speculate that increased pupa weight may be a contributor to the lifespan extension and food intake and fecundity were not to be affected, implying that APS prolongs the lifespan of the silkworm without obvious side-effects.

## Supplementary Materials

The Supplemenantry data can be found online at: www.aginganddisease.org/EN/10.14336/AD.2019.0515.


