# Supplementary material for: Astragalus Polysaccharide Extends Lifespan via Mitigating Endoplasmic Reticulum Stress in the Silkworm, Bombyx mori
Source: Aging Dis. 2019 Dec 1;10(6):1187–98. doi: 10.14336/AD.2019.0515 (PMC6844597; doi:10.14336/AD.2019.0515)
Supplement: Supplementary file 1 [file AD-10-6-1187-s.pdf]

SUPPLEMENTARY DATA

Astragalus Polysaccharide Extends Lifespan via Mitigating Endoplasmic Reticulum Stress in the Silkworm, *Bombyx mori*

Jiangbo Song[#], Min Chen[#], Zhiquan Li, Jianfei Zhang, Hai Hu, Xiaoling Tong[*], Fangyin Dai[1,*]

State Key Laboratory of Silkworm Genome Biology, Key Laboratory for Sericulture Biology and Genetic Breeding, Ministry of Agriculture and Rural Affairs, College of Biotechnology, Southwest University, Chongqing 400716, China

© 2019. Song J et al. Published online at http://www.aginganddisease.org/EN/10.14336/AD.2019.0515

SUPPLEMENTARY DATA

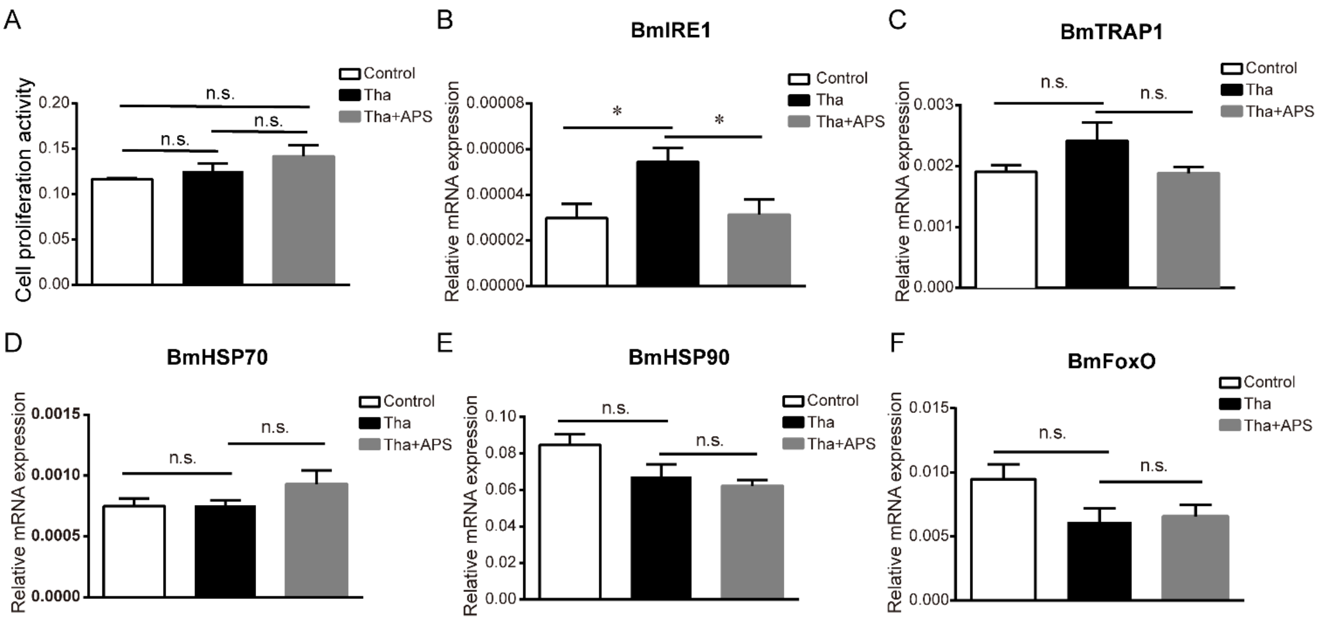

Supplementary Figure 1. Detection of cell proliferation and the response of relative genes after thapsigargin and APS co-treatment.

© 2019. Song J et al. Published online at http://www.aginganddisease.org/EN/10.14336/AD.2019.0515
